# Bismuth-coated 80S15C bioactive glass scaffolds for photothermal antitumor therapy and bone regeneration

**DOI:** 10.3389/fbioe.2022.1098923

**Published:** 2023-01-12

**Authors:** Jianhang Du, Huifeng Ding, Shengyang Fu, Dejian Li, Bin Yu

**Affiliations:** ^1^ Department of Orthopedics, Shanghai Public Health Clinical Center, Fudan University, Shanghai, China; ^2^ Department of Orthopedics, Shanghai Pudong Hospital, Fudan University Pudong Medical Center, Shanghai, China; ^3^ Center for Translational Neurodegeneration and Regenerative Therapy, Tongji Hospital, Tongji University School of Medicine, Shanghai, China

**Keywords:** bismuth, bioactive glass, photothermal antitumor, bone regeneration, 3D printing

## Abstract

**Background:** Malignant bone tumors usually occur in young people and have a high mortality and disability rate. Surgical excision commonly results in residual bone tumor cells and large bone defects, and conventional radiotherapy and chemotherapy may cause significant side effects. In this study, a bifunctional Bi-BG scaffold for near-infrared (NIR)-activated photothermal ablation of bone tumors and enhanced bone defect regeneration is fabricated.

**Methods:** In this study, we prepared the Bi-BG scaffold by *in-situ* generation of NIR-absorbing Bi coating on the surface of a 3D-printing bioactive glass (BG) scaffold. SEM was used to analyze the morphological changes of the scaffolds. In addition, the temperature variation was imaged and recorded under 808 nm NIR laser irradiation in real time by an infrared thermal imaging system. Then, the proliferation of rat bone mesenchymal stem cells (rBMSCs) and Saos-2 on the scaffolds was examined by CCK-8 assay. ALP activity assay and RT-PCR were performed to test the osteogenic capacity. For *in vivo* experiments, the nude rat tumor-forming and rat calvarial defect models were established. At 8 weeks after surgery, micro-CT, and histological staining were performed on harvested calvarial samples.

**Results:** The Bi-BG scaffolds have outstanding photothermal performance under the irradiation of 808 nm NIR at different power densities, while no photothermal effects are observed for pure BG scaffolds. The photothermal temperature of the Bi-BG scaffold can be effectively regulated in the range 26–100°C by controlling the NIR power density and irradiation duration. Bi-BG scaffolds not only significantly induces more than 95% of osteosarcoma cell death (Saos-2) *in vitro*, but also effectively inhibit the growth of bone tumors *in vivo*. Furthermore, they exhibit excellent capability in promoting osteogenic differentiation of rBMSCs and finally enhance new bone formation in the calvarial defects of rats.

**Conclusion:** The Bi-BG scaffolds have bifunctional properties of photothermal antitumor therapy and bone regeneration, which offers an effective method to ablate malignant bone tumors based on photothermal effect.

## 1 Introduction

Primary malignant bone tumors such as osteosarcoma and Ewing sarcoma are most common in the adolescent population with a high mortality and disability rate. They can cause large bone defects after surgical treatment. Some bone tumors are still not completely resectable, and therefore are usually treated with post-operative chemotherapy such as metastatic tumors in the spine. However, the lack of targeting of chemotherapy drugs is associated with serious complications such as infection due to bone marrow suppression, femoral head necrosis and nephrotoxicity (Djunic et al., 2011). Long-term chemotherapy can also make tumor cells resistant to drugs. Radiotherapy as a local treatment has limited indications, particularly for spinal tumors where it is contraindicated. It is therefore a great challenge to prepare biomaterials that combine the treatment of bone tumors with bone defect repair.

In recent years, there has been an increasing interest in photothermal therapy (PTT) to kill tumor cells. When a photothermal material absorbs light in the infrared band, the electrons move from the ground state to the excited state and then release energy through non-radiative decay, causing an increase in kinetic energy to heat the local environment of the material (Cao et al., 2013; Li et al., 2013). Photothermal therapy has been considered a very promising method of tumor treatment because it is localized and avoids damage to normal tissue in non-treated areas (Liu et al., 2009; Von Maltzahn et al., 2009; Choi et al., 2011; Melancon et al., 2011).PTT employs photo-absorbing agents to generate heat from light, thus inducing hyperthermia in tumor sites as well as causing protein denaturation and cell membrane disruption, finally resulting in cell death (Cheng et al., 2014a). As effective newborn PTT agents, Bismuth (Bi)-based nanoparticles (NPs) have been intensely investigated in recent years. So far, most research on PTT agents focus on Bi_2_Se_3_ (Li et al., 2016; Xie et al., 2016), Bi_2_S_3_ (Wang et al., 2016; Xiao et al., 2016), Cu_3_BiS_3_ (Yang et al., 2015; Liu et al., 2016), and Bi NPs (Li et al., 2017; Yu X et al., 2017). The photothermal conversion efficiency (*η*) of these Bi-based NPs is essentially above 30% under NIR laser irradiation. Among them, pure Bi NPs have been demonstrated to exhibit excellent physiological stability, biocompatibility, and extended circulating half-life (Li et al., 2017; Yu X et al., 2017). Due to their strong NIR absorbance as well as the high photothermal conversion efficiency and conversion stability, highly effective *in vivo* photothermal ablation of tumors has been realized under NIR irradiation, without noticeable toxicity (Li et al., 2017; Yu X et al., 2017). Moreover, Bismuth is also used in clinic mainly for the treatment of *H. pylori* infection. It is absorbed orally by the body and distributed primarily to the kidneys, brain, liver and bones. Although it easily forms insoluble precipitates in the stomach, less than .5% is absorbed by the body. Thus, small doses of bismuth rarely cause significant toxic side effects (Leussink et al., 2001).

Bioactive glass is an artificial bone that promotes osteogenesis and provides strong biomechanical properties, while the material is biodegradable. Its main functional elements are Ca, Si, P, and Na. BG is widely used as a bone repair material due to its ability to form a hydroxyapatite layer *in vivo* and to bind biologically to bone tissue (Hench, 1991; Ohtsuki et al., 1992; Hench et al., 1971). In addition, Ca^2+^ and Si^4+^ in bioactive glass promote osteoblast proliferation and differentiation (Gough et al., 2004; Valerio et al., 2004). Yan used a novel method to prepare 80S15C bioactive glass with pores of 5–20 nm, a pore volume of .4 cm^3^/g and a surface area of 300 m^2^/g (Yan et al., 2006). Zhu et al. (2008) found that 80S15C (a relatively low calcium content) has better biological activity *in vitro* compared to convention; ; al bioactive glass. Our previous study found that 80S15C-coated artificial ligaments had a beneficial effect on tendon-bone healing (Yu B et al., 2017). Therefore, we used 80S15C bioactive glass as a substrate for tumorous bone defects.

**FIGURE 1:**
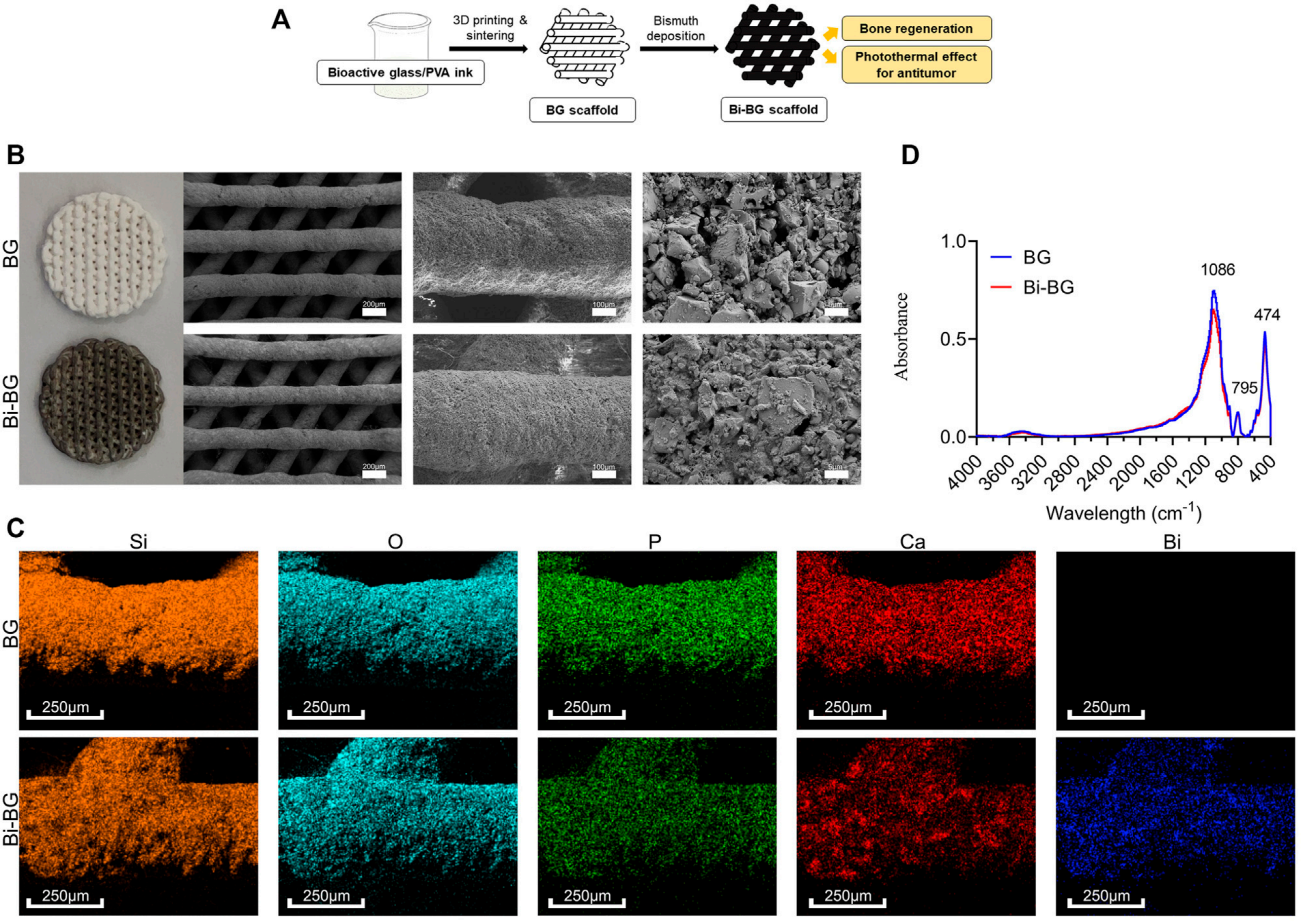
Fabrication and characterizations of BG and Bi-BG scaffolds. **(A)** Schematic illustration for the formation of bifunctional Bi-BG scaffolds. **(B)** Photograph and SEM images of 3D printed pure BG and Bi-BG scaffolds; **(C)** Elemental mapping images (Si, O, P, Ca, and Bi elements) of BG and Bi-BG scaffolds; **(D)** FTIR patterns of the scaffolds.

To our knowledge, no such bioactive scaffold has been designed with both photothermal anti-tumor effects and osteogenic activity. Therefore, the aim of this study was to design and fabricate a bifunctional scaffold to kill osteosarcoma cells and repair bone defects, making it possible to treat large bone defects caused by surgical resection of bone tumors. In this study, BG scaffolds were firstly prepared by three-dimensional (3D) printing, and Bi was modified to the surface of as-printed scaffolds afterwards (Figure 1A). The effects of NIR laser power and irradiation duration on the photothermal effect of scaffolds were systematically investigated. Then, we evaluated the photothermal antitumor effects of Bi-BG scaffolds *in vitro* and *in vivo*. Furthermore, their osteogenic capacity was investigated *in vitro* and *in vivo*.

## 2 Materials and methods

### 2.1 Materials

Tetraethyl orthosilicate (TEOS, 98%), triethylphosphate (TEP, 99%), ethanol, calcium nitrate (Ca(NO_3_)_24_H_2_O, 99%), sodium borohydride, and bismuth nitrate were purchased from Sinopharm Chemical Reagent (China). Polyvinyl alcohol (PVA, Mn = 70–90 k) was purchased from Sigma-Aldrich. All chemicals were used without further purification.

### 2.2 Fabrication and characterizations of Bi-coated 80S15C bioactive glass (Bi-BG) scaffolds

The 80S15C BG powder was prepared by the evaporation-induced self-assembly (EISA) method. Firstly, ethyl orthosilicate (TEOS, 53.6 g), Ca(NO_3_)_2_ - 4 H_2_O (11.2 g), triethyl phosphate (TEP, 5.84 g) and HCl (.5 M, 8 g) were added to ethanol (480 g) and stirred for 24 h. The resulting solution was poured into a glass Petri dish for evaporation-induced self-assembly at room temperature for 7 days. Subsequently, it is dried in an oven at 60°C for 48 h to obtain BG powder.

In this experiment, an aqueous solution of 10 wt% PVA was used as the adhesive agent for the printing paste preparation. Firstly, 5 g of PVA particles were added to a container containing 45 g of deionized water and stirred for 2 h at room temperature to ensure that the PVA particles were sufficiently swollen to facilitate dissolution. The container was then placed in a water bath whose temperature was gradually increased to 92°C, and stirred until completely dissolved. Finally, allow the PVA solution to cool and reserve for use.

The synthesized BG powder is ground and sieved. Then it was added to the aqueous PVA solution and mixed thoroughly to obtain printable BG/PVA slurry (W_BG_: W_PVA_ = 1.2:1). The prepared slurry was then transferred into a syringe to print. The needle for printing has an internal diameter of 400 μm, a syringe temperature of 25°C, a pressure of 2–4 bar and a printing speed of 4–8 mm/s. The piston of the syringe squeezed the slurry out of the needle to form a fibrous form, and the final BG/PVA scaffold was printed by layer-by-layer stacking.

BG and Bi-BG scaffolds were viewed by optical microscope. Scanning electron microscopy (SEM) was carried out with a ZEISS Sigma 300 field emission scanning electron microscope with mapping analysis. SEM images and element mapping were monitored on a SU8220 microscope (HITACHI, Japan). FTIR were measured by Thermo Scientific Nicolet iS10 in the range of 4,000–400 cm^−1^.

### 2.3 *In Vitro* photothermal effect of Bi-BG scaffolds

To evaluate the photothermal property, the Bi-BG scaffolds were placed in a 48-well culture plate, irradiated by the 808 nm NIR laser (LDT-808, Shanghai Connect Fiber Optics Company) for 5 min, and the temperature variation was imaged and recorded in real time by an infrared thermal imaging system (223S, Fotirc). Different power density of the light was applied, including low (1 W/cm^2^), medium (2 W/cm^2^), and high (3 W/cm^2^). The photothermal effect of the scaffolds in wet condition (PBS) was also evaluated for 10 min. Finally, the photothermal conversion curve over time was plotted using FLIR R&D software.

### 2.4 *In Vitro* photothermal antitumor effect of Bi-BG scaffolds

All scaffolds were sterilized with ethylene oxide and prepared for use. To evaluate the *in vitro* photothermal anti-tumor effect of the Bi-BG scaffold, Saos-2 cells were inoculated in 48-well plates at a density of 1 × 10^4^ cells per well for 24 h (*n* = 4). For the laser group, the scaffold was placed in the well plate and irradiated with an 808 nm laser (3 W/cm^2^) for 10 min, then they were removed. For the no-laser group, the scaffolds were placed in the well plate for 10 min and then removed. Saos-2 cells were then incubated for a further 12 h. The proliferation of Saos-2 cells with the scaffolds was tested using a standard CCK-8 method. The effect of different irradiation time on saos-2 cell viability were further evaluated. The cells were co-cultured with the Bi-BG scaffold for 24 h and then irradiated with a NIR laser at a power density of 3 W/cm^−2^ for 10, 20, and 30 min respectively.

Live/dead cell staining is used to observe the viability of tumor cells after photothermal treatment. Firstly, Saos-2 cells were seeded on round coverslip (*n* = 2). After the cells had grown all over the coverslip, the scaffolds were placed slightly into the well plate. For the laser groups, the scaffolds were exposed to a NIR laser at 3 W/cm^2^ for 15 min and then removed. For the no-laser groups, the scaffolds were placed in the well plate for 15 min and then removed. After washed three times with PBS, the cells were stained for 20 min with Calcein AM (Life Technologies, United States) and Ethidium homodimer-1 (Life Technologies, United States). Finally, the status and activity of Saos-2 cells on the coverslip were observed using a single photon laser confocal microscopic imaging system (Leica TCS SP8, Germany).

### 2.5 *In Vivo* photothermal antitumor effect of Bi-BG scaffolds

A total of 24 female nude mice (Balb/c) of 4–6 weeks were used for this study. Saos-2 cell suspension (5 × 10^6^ cells/each) was injected subcutaneously into the back of nude mice. When the subcutaneous tumor volume reached about 114 mm^3^, the nude mice were randomly divided into four groups: BG group, BG + laser group, Bi-BG group and Bi-BG + laser group (*n* = 6). A small incision was carefully made in the skin at the tumor margin and the scaffold (8 mm × 1.5 mm) was inserted into the center of the tumor. The wound was then closed with surgical sutures. After 24 h, the mice in the BG laser and Bi-BG laser groups were anesthetized and irradiated with an 808 nm NIR laser (3 W/cm^−2^) for 10 min every 3 days. The real-time temperature and *in situ* thermal images at tumor sites of the mice were monitored using an infrared thermographic camera. The tumor volumes of all mice were recorded every other day. Tumor volume (V) = (tumor length) × (tumor width)^2^/2—scaffold volume; scaffold volume = 60 mm^3^. V_0_ is the initial tumor volume-scaffold volume at day 0. The relative tumor size = V/V_0_.

Finally, tumor specimens were harvested after 2 weeks for hematoxylin and eosin (HE). Tumor cell necrosis rate (TCNR) was calculated according to the formula, TCNR = (1—N/M) × 100%, where M is the mean cell survival rate of normal tumor tissue without treatment and N is the cell survival rate of the scaffold-treated samples. To further verify the *in vivo* biosafety of BG and BI-BG scaffolds, the major organs (heart, liver, spleen, lungs, and kidneys) of all mice were collected for HE staining.

### 2.6 *In Vitro* osteogenic differentiation of rBMSCs on Bi-BG scaffolds

rBMSCs were used in the following experiments. Firstly, the proliferation of rBMSCs on the scaffold surface was quantitatively assessed using CCK-8 method. Bi and Bi-BG scaffolds were sterilized and placed in 24-well plates, then rBMSCs were inoculated onto the surface of the scaffolds at a density of 1 × 10^4^ per well. Osteogenic differentiation was induced by replacing the complete medium with osteogenic induction solution for 7 and 14 days respectively. At the established time points, 200 μL of cell lysis solution was added to each well and the supernatant was collected after 5 min of lysis on ice. The ALP activity of protein samples was measured at 405 nm according to the instructions (Beyotime, Shanghai). Finally, the protein concentration of the samples was determined by the bicinchoninic acid (BCA) method to standardize the ALP activity.

All scaffolds were sterilized and placed in 6-well plates. 2 × 10^5^ rBMSCs of forth generation were added to each well and the medium was changed every other day. The expression levels of osteogenesis-related genes (OCN, BSP, BMP2, OPN, ALP) were measured by RT-qPCR after 7 and 14 days of incubation, respectively. At the time point of the assay, cells were lysed with 1 mL of Trizol reagent for 15 min to extract RNA after removal of the medium. The RNA was then reverse transcribed into cDNA using iScript cDNA Synthesis kit. Subsequently, the Ct values of the osteogenic genes were detected on a PCR instrument using the SYBR Green PCR Master Mix Kit, with the GAPDH gene as an internal reference. Finally, the expression levels of the osteogenesis-related genes were calculated by the ΔΔCt method.

### 2.7 *In Vivo* bone regeneration of Bi-BG scaffolds

All experimental procedures described in this study were approved by the Animal Ethics Committee of Fudan University Pudong Medical Center. Eighteen 7-week-old male Sprague Dawley (SD) rats were used for surgery and randomly divided into three groups as follows: 1) Blank control (*n* = 6); 2) BG (*n* = 6), and 3) Bi-BG (*n* = 6). Anesthesia was administered to rats by intraperitoneal injection of 3% (w/v) sodium pentobarbital (30 mg/kg). A 1.5-cm sagittal incision is made in the scalp along the median line: the skin and subcutaneous tissues of the rat are incised in sequence, followed by exposure of the skull *via* blunt dissection. Two 5-mm cranial defects were created using an electric torus drill, and then the scaffold material was placed in the defects. Finally, the incision was closed by suturing the periosteum and skin. Each rat was given penicillin intramuscularly for 3 days after surgery. All animals were euthanized by injection of an overdose of sodium pentobarbital 8 weeks after surgery for the following experiments.

All rats were executed 8 weeks after implantation of the scaffolds. The skull was dissected and the surrounding muscles and soft tissues were removed. The newly harvested skull was then scanned using micro-CT (Skyscan1176, Kontich, Belgium) with a resolution of 18 μm to assess the formation of new bone. Finally, 3D images were reconstructed using 3D Creator software to determine bone volume versus total bone volume (BV/TV) and local bone mineral density (BMD). The removed skull was placed in 4% paraformaldehyde for 24 h and then decalcified with 5% nitric acid for 1 week, with the solution changed every other day. This was followed by consecutive dehydration in gradient ethanol (70%–100%) and toluene. The dehydrated samples were embedded using paraffin wax and hardened into paraffin blocks. A slicer (HM 325, Thermo Fisher Scientific) was then used to cut the paraffin blocks to obtain 10 μm thick sections in the sagittal direction. After deparaffinization and hydration, the sections were stained with HE and Masson. Finally, the sections were observed under a microscope and images were captured using a histological digital scanning system (NanoZoomerS210, Hamamatsu) to assess the repair of the skull defect.

### 2.8 Statistical analyses

All data were collected from three parallel samples and statistically analyzed using Graphpad Prism 10 software. The experimental results are expressed as mean ± standard deviation (mean ± SD). Multiple groups were statistically analyzed by one-way analysis of variance (ANOVA) and Student-Newman-Keuls post-hoc tests. *p* < .05 indicated that there is a statistical difference.

## 3 Results and discussion

### 3.1 Preparation and characterization of Bi-BG scaffolds

BG scaffolds were prepared from BG powders *via* 3D printing, which is a rapid and concise technique for complex structures forming. We chose the structure with macro-pores (400 μm) (Figure 1b), which has been proved to facilitate cell attachment and migration, nutrient transport, blood vessel growth, and bone formation (Zhu et al., 2015; Rustom et al., 2016). And the crystals could be found on the surface of the scaffolds after sintering. The main elements on scaffolds were Si, O, P, and Ca that were the BG powder composition, and the surface structures seemed to be no different between BG scaffolds after Bi modification (Figure 1b). However, Bi could be detected *via* element analysis with a uniform distribution (Figure 1c). There was no morphological difference that could be attributed to the limited Bi modification on the scaffolds. BG and Bi-BG scaffolds showed similar absorbance peaks evaluated by FTIR (Figure 1d), where the peak on 474 cm^−1^ was the bending vibration absorption peak of Si-O, 795 cm^−1^ was the symmetric stretching vibration peak of Si-O, and 1,086 and 795 cm^−1^ were the asymmetric stretching vibration peak of Si-O-Si. These peaks conformed to the main composition of Si and O in both BG and Bi-BG scaffolds.

As a biocompatible photothermal conversion agent (Chen et al., 2019), Bi has been introduced to surface-modify 3D printed bioactive glass scaffolds to provide them with excellent photothermal properties and enhanced osteogenesis. In this study, we combined 3D printing technology and Bi surface modification to fabricate Bi-BG scaffolds. 3D printing enables good control of the macroporous size and geometry of 80Si15C scaffolds through computer-aided design (CAD). The Bi-BG scaffold can therefore be shaped specifically according to the individual requirements of the patients, compared to other techniques. Bi coatings are easily and firmly deposited on the surface of 3D printed BG scaffolds by direct solution immersion and *in-situ* reduction, which do not damage their macroporous structure. Finally, we have successfully prepared a bifunctional Bi-BG scaffold for bone tumor treatment and the repair of large bone defects.

### 3.2 Photothermal effect of Bi-BG scaffolds

**FIGURE 2:**
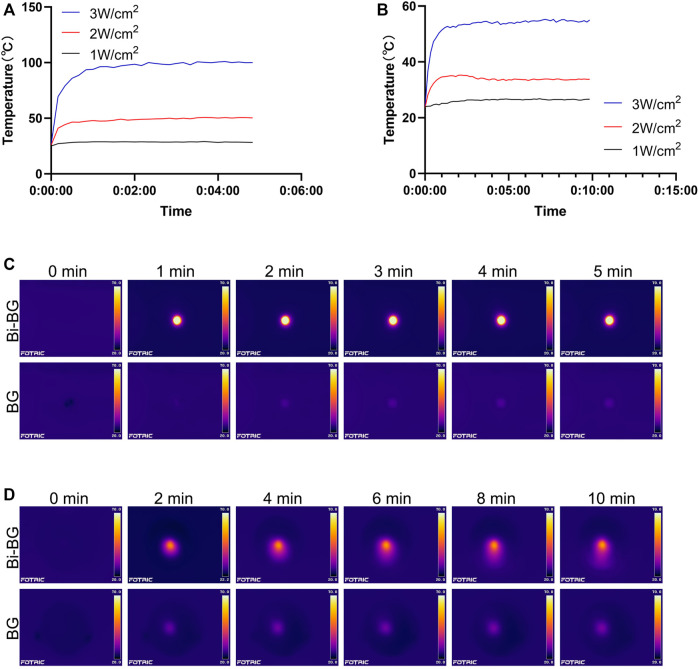
Photothermal effect of Bi-BG scaffolds. Heating curves of Bi-BG scaffolds with different laser power irradiation in **(A)** dry state and in **(B)** PBS; infrared thermal images of Bi-BG and BG scaffolds in **(C)** dry state and in **(D)** PBS under 808 nm laser irradiation.

The photothermal effect of Bi-BG scaffolds was evaluated *in vitro*. It could be found that the Bi-BG scaffolds could induce a temperature rise under NIR irradiation, and the temperature rise could be regulated by laser power (Figure 2A), while the temperature of BG scaffolds stayed at room temperature. Under the high power laser irradiation, the temperature of the Bi-BG scaffolds rises rapidly from room temperature to 100°C in 60 s and remains stable at around 100°C thereafter. In infrared thermal images, the Bi-BG scaffolds showed a more obvious thermal signal compared with BG scaffolds (Figure 2C). Furthermore, when the Bi-BG scaffolds were in wet condition, the temperature could also be raised under NIR irradiation (Figure 2B), but was much lower (53°C) than that in the dry condition (100°C). However, Bi-BG scaffolds still showed a brighter thermal signal than that of BG scaffolds (Figure 2D). These results demonstrated that the Bi modification endowed the excellent photothermal effect to BG scaffolds.

There are many Bi-related photothermal materials, and we have chosen pure Bi NPs because their photothermal conversion efficiency is comparable to, or even higher than, that of other Bi-related materials (Cheng and Zhang, 2018). Studies have shown that Bi not only has strong absorption of NIR laser (808 nm) (Peng et al., 2009), but also efficiently converts it into thermal energy (Peng et al., 2011). Pure Bi NPs can be used as a photothermal agent in cancer therapy because its photothermal effect penetrates the skin and is non-invasive and harmless (Mohan, 2010; Yu X et al., 2017; Li et al., 2017). However, there is no report focusing on the origin of photothermal performance of Bi NPs. As semimetal, Bi NPs not only exhibit the surface plasmon resonances (SPR) effect (Sun et al., 2015; Zhao et al., 2016) which is similar to noble metals, but also have narrow bandgap (Qi et al., 2008; Velasco-Arias et al., 2012) like semiconductors. As a result, we presume that its origin of the photothermal property may be similar with that of Bi_2_Se_3_ NPs. However, the photothermal mechanism of pure Bi NPs needs to be further investigated, beneficial for improving their photothermal efficacy. In this study, we have successfully fabricated a bifunctional Bi-BG scaffold with excellent photothermal properties to raise the ambient temperature to kill osteosarcoma cells. In addition, the Bi-BG scaffold temperature can be efficiently regulated by controlling the laser power and irradiation time over a wide range from room temperature to 100°C. However, the photothermal effect was found to be significantly lower in wet conditions than in dry conditions. One reason is that the PBS solution in which the scaffold is immersed absorbs some of the NIR and thermal energy. The other is that wet conditions may reduce the thermal conductivity of the Bi-BG scaffold.

### 3.3 Photothermal effect of Bi-BG scaffolds for killing bone-tumor cells

**FIGURE 3:**
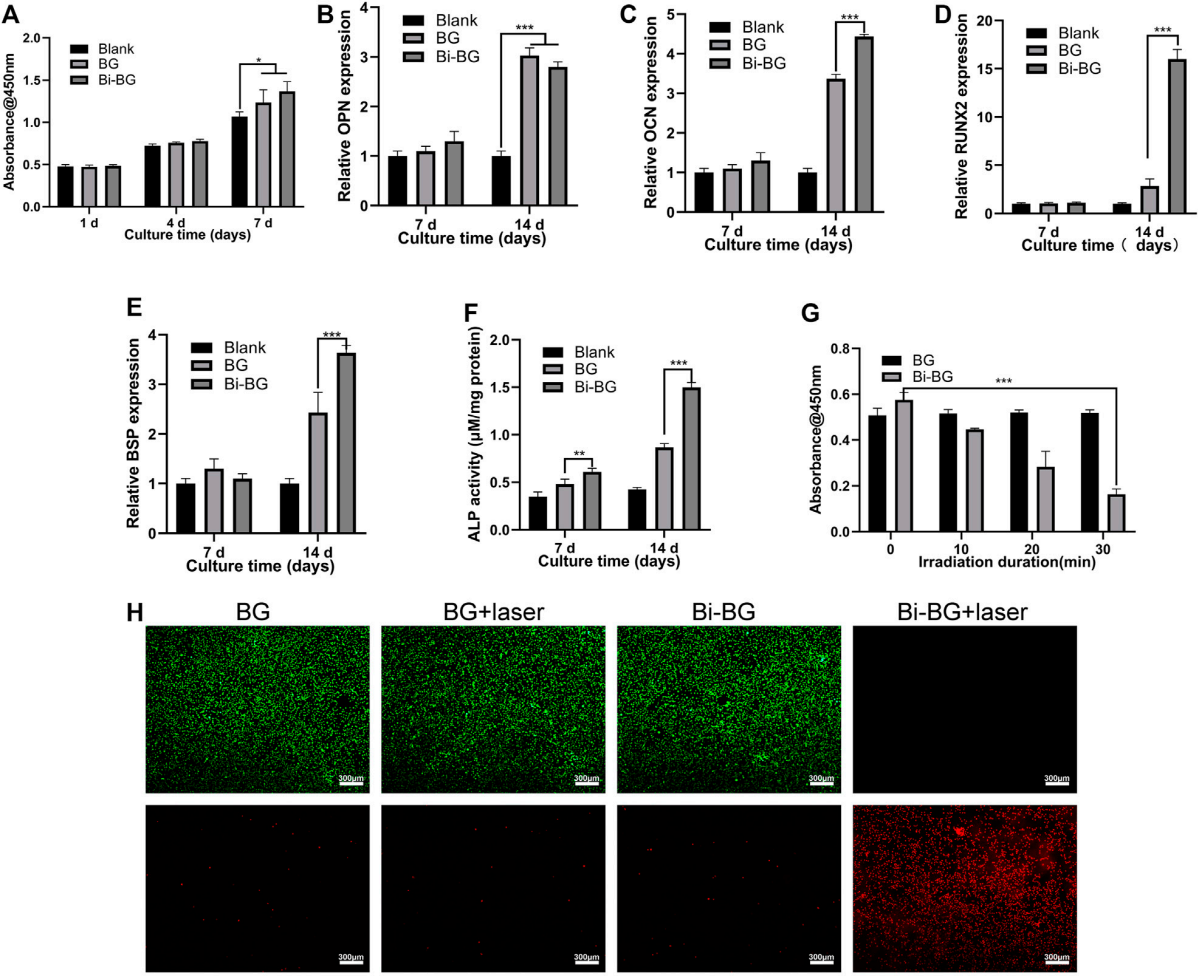
*In vitro* osteogenic and tumor-killing assays. **(A)** Proliferation of rBMSCs incubated on BG and Bi-BG scaffolds for 1, 4, and 7 days; **(B)** osteogenic expression of OPN, **(C)** OCN, **(D)** Runx2, and **(E)** BSP, and **(F)** ALP activity of rBMSCs on the scaffolds for 7 and 14 days (*n* = 3). **(G)** Saos-2 cell viability of on BG and Bi-BG scaffolds treated with different irradiation duration; **(H)** fluorescent images of Saos-2 cells in four groups after 15 min of laser irradiation at 3 W/cm^2^. Green represents live cells and red dead (*, **, and *** indicate *p* < .05, *p* < .01, and *p* < .001, respectively).

We investigated the killing effect of the Bi-BG scaffolds on osteosarcoma Saos-2 cells under laser irradiation. The viability of osteosarcoma cells was found to decrease sharply with increasing irradiation time (Figure 3G). After 30 min of laser irradiation, the viability of saos-2 cells dropped to a minimum of .164. However, the cellular viability of the BG groups remained largely unchanged after different irradiation durations. In addition, we performed live-dead cell staining after laser irradiation to further assess the tumor-killing effect of the Bi-BG scaffolds. It can be observed that the Saos-2 cells in the Bi-BG group underwent extensive death after laser irradiation, whereas the Saos-2 cells in the other groups grew well and no significant necrosis was seen (Figure 3H). The above results showed that irradiation time plays a key role in killing osteosarcoma cells. The viability of Saos-2 cells was reduced by 71.5% within 30 min, indicating that the Bi-BG scaffolds have a highly effective photothermal osteosarcoma-killing effect.

### 3.4 *In Vivo* photothermal therapy of tumor tissue by Bi-BG scaffolds

**FIGURE 4:**
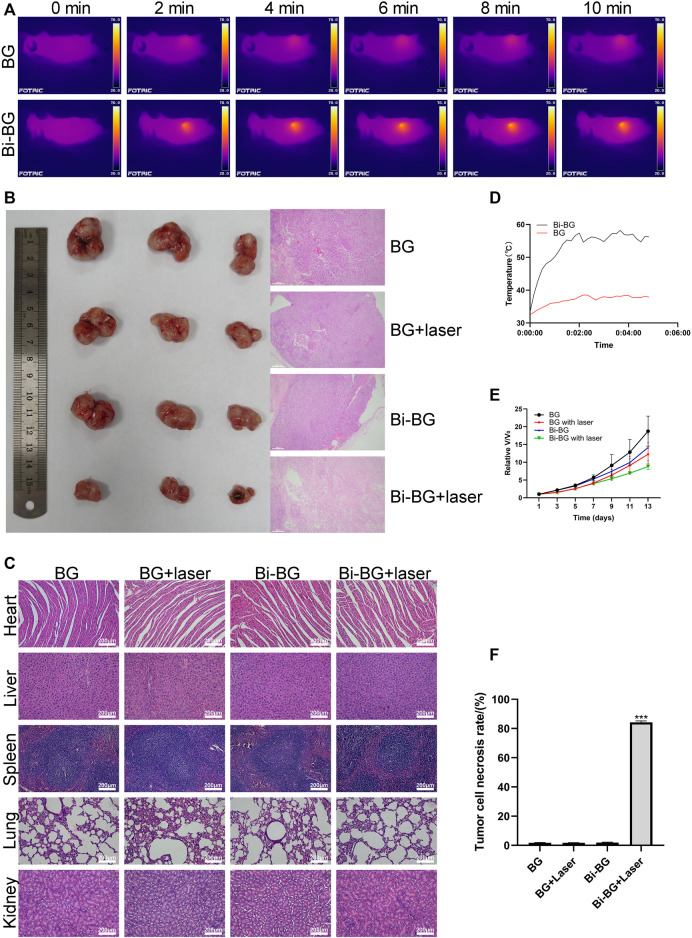
*In vivo* photothermal antitumor effect of Bi-BG scaffolds. **(A)** IR thermal images, **(B)** optical and H&E staining images of tumor in four groups at day 13, **(C)** H&E staining images of major organs from the four groups (scale bar: 100 μm). **(D)** Heating curve of BG and Bi-BG scaffolds in nude mice under 808 nm laser irradiation, **(E)** relative tumor volume change with increasing days of the groups, and **(F)** the tumor cell necrosis rate (TCNR) of four groups.

To evaluate the photothermal anti-tumor effect of the Bi-BG scaffolds *in vivo*, we used Saos-2 osteosarcoma cells to form tumors subcutaneously in nude mice. When the subcutaneous tumor had reached a volume of 114 mm^3^, the scaffolds were implanted in it and irradiated with NIR light. As shown in Figures 4A, D, the temperature of the tumor center in the Bi-BG group rises rapidly *in vivo* after irradiation with NIR light and remains above 50°C. Hyperthermia (>50°C) within the tumor causes not only late progressive apoptosis of the tumor cells, but also cell necrosis due to irreversible protein denaturation and cell membrane damage (Nikfarjam et al., 2005; Kirui et al., 2010; Lee et al., 2010; Markovic et al., 2011). In addition, the excellent photothermal performance and thermal conductivity of Bi-BG scaffolds kept the temperature of the tumor tissue edge above 45°C, which induces a state of prolonged cell inactivation (Jaque et al., 2014), thus resulting in tumor growth inhibition. However, the temperature of BG scaffolds changes little after irradiation and remains at room temperature due to their poor NIR absorption efficiency and photothermal conversion properties. It can be found that after laser irradiation, the tumor growth rate of the Bi-BG group was significantly slowed down compared to the other three groups (Figure 4E). In addition, the tumors were removed at day 13 after five times of irradiation and it could be seen that the tumor volume of the Bi-BG laser group was the smallest of all four groups. Finally, the HE staining results showed that a large number of tumor nuclei around the scaffold in the Bi-BG laser group were wrinkled and disappeared, while the tumor tissue in the other three groups was normal (Figure 4B). Quantitative analysis revealed that the Bi-BG group had the highest necrosis rate of tumor tissue around the scaffold reaching 85% (Figure 4F). The above results showed that the fabricated Bi-BG scaffold has outstanding photothermal properties and can efficiently kill saos-2 cells *in vitro* and ablate osteosarcoma tissue *in vivo*. Photothermal therapy can selectively kill tumor cells without damaging normal tissue when not exposed to laser radiation (Cheng et al., 2014a). In this study, “selectivity” involves two aspects. One is being able to control where the Bi-BG scaffold is implanted. In our work, the Bi-BG scaffold was implanted into the center of the tumor tissue. The other one is the ability to control the emission of the NIR laser and the tumor site to be irradiated. Recently, microneedles (MNs) have been combined with various therapy strategies including photodynamic therapy (PDT) and PTT to treat many diseases due to its improved selectivity, and minimal invasiveness and side effects (Zhi et al., 2020). Finally, no significant morphological or pathological changes were found in the major organs of all treatment groups (Figure 4C). Therefore, the Bi-BG scaffolds could effectively inhibit bone tumor growth under NIR laser irradiation with excellent biocompatibility.

### 3.5 *In Vitro* osteogenic differentiation of rBMSCs for Bi-BG scaffolds

The proliferation of rBMSCs on Bi-BG scaffolds was tested at 1, 4, and 7 days. As shown in Figure 3A, the proliferation viability of rBMSCs of each group increased over time. At day 7, the proliferation viability of BG and Bi-BG groups was significantly higher than that of blank group. More importantly, the proliferation viability of Bi-BG group was statistically higher than that of BG group. These results indicate that the Bi coating on the surface of BG scaffold significantly promoted rBMSC proliferation, which is consistent with the previous studies (Pazarçeviren et al., 2018; Huang et al., 2020). To assess the osteogenic differentiation on Bi-BG scaffolds, the alkaline phosphatase (ALP) activity of rBMSCs was measured at 7 and 14 days of culture. As shown in Figure 3F, the ALP activity of rBMSCs in BG and Bi-BG groups was higher that of blank group. And compared to BG group, the ALP activity of cells on Bi-BG scaffold was significantly higher. In addition, the expression of osteogenic-related genes (OCN, OPN, BSP, RUNX2) (Ivaska et al., 2004; Alford and Hankenson, 2006) of rBMSCs on the scaffold was measured at 7 and 14 days of culture (Figures 3B–E). There was no statistical difference in the expression levels of osteogenesis-related genes of each group at day 7. At day 14, the expression levels of OCN, BSP and RUNX2 in Bi-BG group were significantly higher than those in BG group, but there was no statistical difference in the expression levels of OPN between the two groups. The results show that both BG and Bi-BG scaffolds have favorable osteogenic capacity, which may be attributed to the large pore structure of 3D printed scaffolds that facilitates the proliferation, adhesion and osteogenic differentiation of mesenchymal stem cells (Rustom et al., 2016). This is consistent with our previous findings (Zhu et al., 2008; Yu B et al., 2017). And Bi coating on the Bi-BG scaffolds significantly promoted osteogenic differentiation of rBMSCs, which is in line with previous research findings (Wang et al., 2018). This could be explained by the good biocompatibility and chemical stability of the Bi coating (Chiang et al., 2014; Wang et al., 2018).

### 3.6 *In Vivo* bone regeneration for Bi-BG scaffolds

**FIGURE 5:**
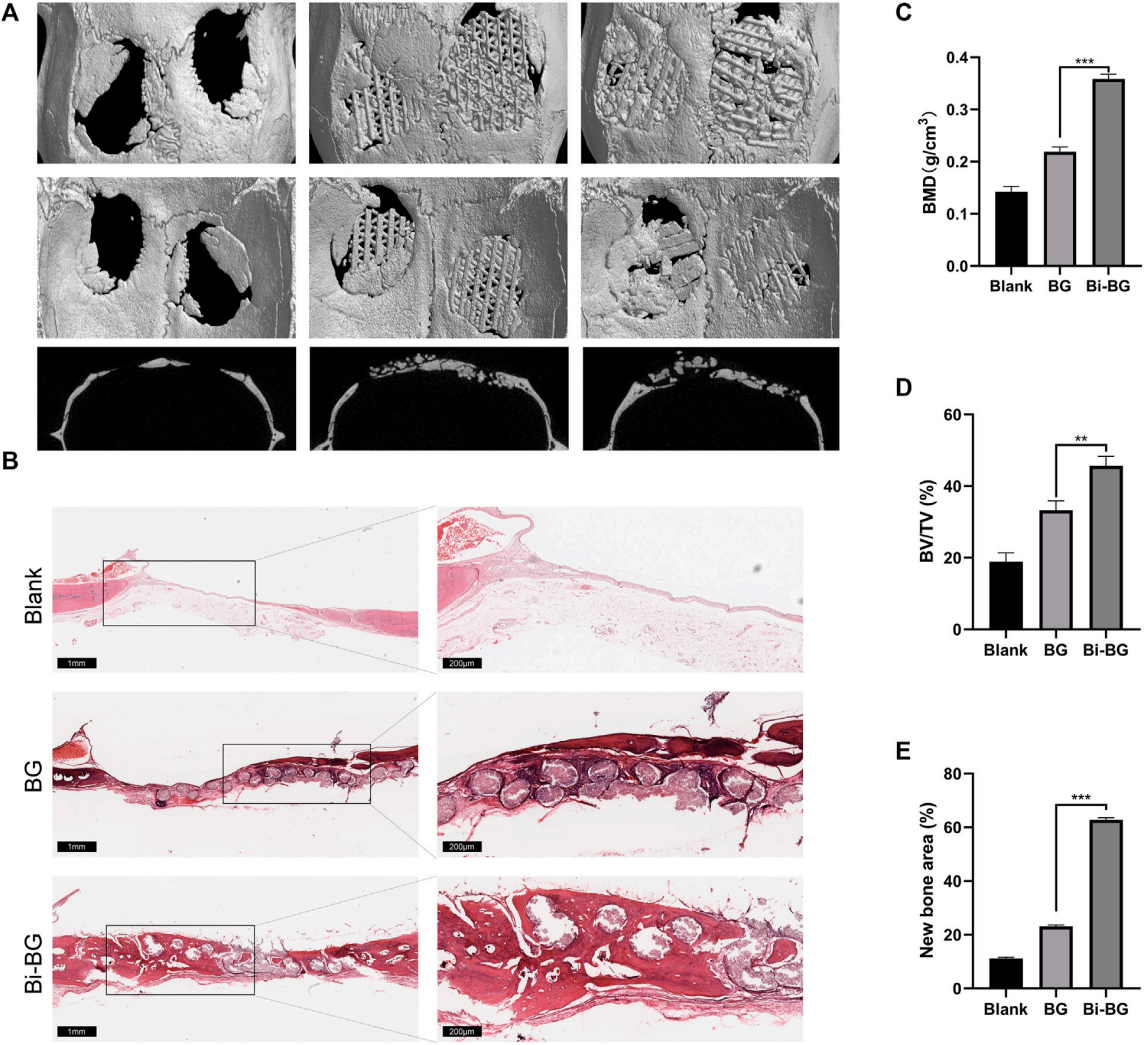
*In vivo* bone regeneration of Bi-BG scaffolds. **(A)** 3D reconstruction and sagittal Micro-CT images of the repaired skulls and **(B)** HE staining images of the blank, BG and Bi-BG groups after 8 weeks’ implantation. **(C)** BMD, **(D)** BV/TV, and **(E)** new bone area of each group.

To evaluate the osteogenic capacity of Bi-BG scaffolds *in vivo*, 3D reconstructions were performed on the harvested rat calvarial specimens. As shown in Figure 5A, the Bi-BG scaffolds were well-integrated with the skull defects of the rats and more bone tissue was formed on the scaffolds than the BG scaffolds. Quantitative analysis revealed that the BV/TV and BMD values of Bi-BG group were significantly higher than those of BG and blank groups after 8 weeks (Figures 5C,D). In addition, the HE staining of the calvaria specimens further demonstrated that large amounts of new bone tissue were formed around the Bi-BG scaffolds (Figure 5B). As shown in Figure 5E, Bi-BG group had a considerably higher amount of new bone than BG group. The above results suggested that the modification of Bi coating on BG scaffolds could further promote the regeneration of bone tissue *in vivo*. The results of *in vivo* experiments further validated that the Bi coating on the surface of the Bi-BG scaffolds improves the osteogenic capacity of the scaffold. However, its specific molecular mechanism remains unclear and further research is needed.

It is well known that for bone tissue engineering, large animal models (at least rabbits) should be used, since they can provide relatively large bone defects (Chen et al., 2013; Dong et al., 2013; Kim et al., 2014; Prosecká et al., 2015). However, no such bone cancer models have so far been available in rabbits or larger animals due to immune rejection. Although bone tumor models can be established using nude mice, their bone size is too small for large bone defects. Therefore, in this study, we evaluated the photothermal anti-tumor effect of the Bi-BG scaffold using tumor-bearing nude mouse models to and their bone repair capacity in rabbits. Notably, the hyperthermia induced by photothermal therapy in clinical practice inevitably has a negative impact on surrounding normal bone cells while killing tumor cells. Under laser irradiation, the temperature of photothermal materials typically rises to 41–47°C or higher (Tian et al., 2013; Cheng et al., 2014b; Zhu et al., 2016). Tumor tissue has been shown to be much less tolerant of hyperthermia than normal cells, with the majority of tumor cells having a thermally lethal temperature of 42–43°C.However, normal cells are able to survive such hyperthermia for long periods of time. This may be due to the high metabolic level and low heat dissipation capacity of the tumor, as well as the acidic interstitial environment (Chu and Dupuy, 2014). The results confirmed that the fabricated Bi-BG scaffolds significantly promote the adhesion, proliferation and differentiation of rBMSCs and new bone formation *in vivo*. Therefore, photothermal therapy with the Bi-BG scaffolds has potential applications in facilitating the repair of large bone defects from surgical resection of bone tumors.

## 4 Conclusion

In conclusion, a bifunctional Bi-BG composite scaffold was successfully prepared by combining 3D printing technology and Bi surface modification, which allows photothermal ablation of bone tumors and enhanced osteogenic capacity. Systematic *in vitro* and *in vivo* evaluation demonstrated that the Bi-BG scaffold exhibited excellent photothermal performance, effectively ablating osteosarcoma cells *in vitro* and inhibiting tumor growth *in vivo* under NIR irradiation. More importantly, the fabricated the Bi-BG scaffold significantly increased new bone formation *in vivo* by promoting osteogenic differentiation of rBMSCs. Our results show that the scaffold can be used for the treatment and repair of large bone defects associated with bone tumors. This work may pave the way for the treatment and repair of tumor-associated tissue defects by developing novel bifunctional scaffolds.

## Data Availability

The original contributions presented in the study are included in the article/Supplementary Material, further inquiries can be directed to the corresponding authors.

## References

[B1] Alford A. I. Hankenson K. D. (2006). Matricellular proteins: Extracellular modulators of bone development, remodeling, and regeneration. Bone 38 (6), 749–757. 10.1016/j.bone.2005.11.017 16412713

[B2] Cao L. Meziani M. J. Sahu S. Sun Y. P. (2013). Photoluminescence properties of graphene versus other carbon nanomaterials. Acc. Chem. Res. 46 (1), 171–180. 10.1021/ar300128j 23092181

[B3] Chen S-H. Lei M. Xie X-H. Zheng L. Z. Yao D. Wang X. L. (2013). PLGA/TCP composite scaffold incorporating bioactive phytomolecule icaritin for enhancement of bone defect repair in rabbits. Acta Biomater. 9 (5), 6711–6722. 10.1016/j.actbio.2013.01.024 23376238

[B4] Chen T. Cen D. Ren Z. Wang Y. Cai X. Huang J. (2019). Bismuth embedded silica nanoparticles loaded with autophagy suppressant to promote photothermal therapy. Biomaterials 221, 119419. 10.1016/j.biomaterials.2019.119419 31421315

[B5] Cheng L. Wang C. Feng L. Yang K. Liu Z. (2014a). Functional nanomaterials for phototherapies of cancer. Chem. Rev. 114 (21), 10869–10939. 10.1021/cr400532z 25260098

[B6] Cheng L. Liu J. Gu X. Gong H. Shi X. Liu T. (2014b). PEGylated WS_2_Nanosheets as a multifunctional theranostic agent for *in vivo* dual-modal CT/photoacoustic imaging guided photothermal therapy. Adv. Mater. 26 (12), 1886–1893. 10.1002/adma.201304497 24375758

[B7] Cheng Y. Zhang H. (2018). Novel bismuth-based nanomaterials used for cancer diagnosis and therapy. Chemistry–A Eur. J. 24 (66), 17405–17418. 10.1002/chem.201801588 29876975

[B8] Chiang T-Y. Wei C-K. Ding S-J. (2014). Effects of bismuth oxide on physicochemical properties and osteogenic activity of dicalcium silicate cements [J]. J. Med. Biol. Eng. 34 (1), 30–35. 10.5405/jmbe.1386

[B9] Choi W. I. Kim J-Y. Kang C. Byeon C. C. Kim Y. H. Tae G. (2011). Tumor regression *in vivo* by photothermal therapy based on gold-nanorod-loaded, functional nanocarriers. ACS Nano 5 (3), 1995–2003. 10.1021/nn103047r 21344891

[B10] Chu K. F. Dupuy D. E. (2014). Thermal ablation of tumours: Biological mechanisms and advances in therapy. Nat. Rev. Cancer 14 (3), 199–208. 10.1038/nrc3672 24561446

[B11] Djunic I. Elezovic I. Marinkovic M. Suvajdzic-Vukovic N. Tomin D. Jankovic G. (2011). Osteolytic lesions marker in multiple myeloma. Med. Oncol. 28 (1), 237–240. 10.1007/s12032-010-9432-4 20127208

[B12] Dong J. Cui G. Bi L. Li J. Lei W. (2013). The mechanical and biological studies of calcium phosphate cement-fibrin glue for bone reconstruction of rabbit femoral defects. Int. J. Nanomedicine 8, 1317–1324. 10.2147/ijn.s42862 23576869 PMC3617789

[B13] Gough J. Notingher I. Hench L. (2004). Osteoblast attachment and mineralized nodule formation on rough and smooth 45S5 bioactive glass monoliths. J. Biomed. Mater. Res. Part A 68 (4), 640–650. 10.1002/jbm.a.20075 14986319

[B14] Hench L. L. (1991). Bioceramics: From concept to clinic. J. Am. Ceram. Soc. 74 (7), 1487–1510. 10.1111/j.1151-2916.1991.tb07132.x

[B15] Hench L. L. Splinter R. J. Allen W. Greenlee T. K. (1971). Bonding mechanisms at the interface of ceramic prosthetic materials. J. Biomed. Mater Res. 5 (6), 117–141. 10.1002/jbm.820050611

[B16] Huang Z. Liu C. Lu Y. Su H. Wang Q. Zheng C. (2020). The treatment effect of highly biocompatible bismuth/strontium/hydroxyapatite/chitosan for osteosarcoma, bacterial and bone defects [J]. Rev. Chim. 71, 111–123. 10.37358/RC.20.6.8176

[B17] Ivaska K. Hentunen T. A. Vaaraniemi J. Ylipahkala H. Pettersson K. Vaananen H. K. (2004). Release of intact and fragmented osteocalcin molecules from bone matrix during bone resorption *in vitro* . J. Biol. Chem. 279 (18), 18361–18369. 10.1074/jbc.M314324200 14970229

[B18] Jaque D. Maestro L. M. Del Rosal B. Haro-Gonzalez P. Benayas A. Plaza J. L. (2014). Nanoparticles for photothermal therapies. nanoscale 6 (16), 9494–9530. 10.1039/c4nr00708e 25030381

[B19] Kim J. Mcbride S. Dean D. D. Sylvia V. L. Doll B. A. Hollinger J. O. (2014). *In vivo*performance of combinations of autograft, demineralized bone matrix, and tricalcium phosphate in a rabbit femoral defect model. Biomed. Mater. 9 (3), 035010. 10.1088/1748-6041/9/3/035010 24784998

[B20] Kirui D. K. Rey D. A. Batt C. A. (2010). Gold hybrid nanoparticles for targeted phototherapy and cancer imaging. Nanotechnology 21 (10), 105105. 10.1088/0957-4484/21/10/105105 20154383

[B21] Lee C. Hong C. Kim H. Kang J. Zheng H. M. (2010). TiO2 nanotubes as a therapeutic agent for cancer thermotherapy. Photochem Photobiol. 86 (4), 981–989. 10.1111/j.1751-1097.2010.00731.x 20408983

[B22] Leussink B. T. Slikkerveer A. Engelbrecht M. R. Van der Voet G. B. Nouwen E. J. De Heer E. (2001). Bismuth overdosing-induced reversible nephropathy in rats. Arch. Toxicol. 74 (12), 745–754. 10.1007/s002040000190 11305776

[B23] Li J-L. Tang B. Yuan B. Sun L. Wang X. G. (2013). A review of optical imaging and therapy using nanosized graphene and graphene oxide. Biomaterials 34 (37), 9519–9534. 10.1016/j.biomaterials.2013.08.066 24034502

[B24] Li Z. Liu J. Hu Y. Fan X. Sun Y. Yu M. (2017). Biocompatible PEGylated bismuth nanocrystals: “All-in-one” theranostic agent with triple-modal imaging and efficient *in vivo* photothermal ablation of tumors. Biomaterials 141, 284–295. 10.1016/j.biomaterials.2017.06.033 28709019

[B25] Li Z. Liu J. Hu Y. Howard K. A. Fan X. Chang M. (2016). Multimodal imaging-guided antitumor photothermal therapy and drug delivery using bismuth selenide spherical sponge. ACS Nano 10 (10), 9646–9658. 10.1021/acsnano.6b05427 27689234

[B26] Liu J. Wang P. Zhang X. Wang L. Wang D. Gu Z. (2016). Rapid degradation and high renal clearance of Cu3BiS3 nanodots for efficient cancer diagnosis and photothermal therapy *in vivo* . ACS Nano 10 (4), 4587–4598. 10.1021/acsnano.6b00745 27014806

[B27] Liu Z. Tabakman S. M. Chen Z. Dai H. (2009). Preparation of carbon nanotube bioconjugates for biomedical applications. Nat. Protoc. 4 (9), 1372–1381. 10.1038/nprot.2009.146 19730421 PMC2853228

[B28] Markovic Z. M. Harhaji-Trajkovic L. M. Todorovic-Markovic B. M. Kepic D. P. Arsikin K. M. Jovanovic S. P. (2011). *In vitro* comparison of the photothermal anticancer activity of graphene nanoparticles and carbon nanotubes. Biomaterials 32 (4), 1121–1129. 10.1016/j.biomaterials.2010.10.030 21071083

[B29] Melancon M. P. Zhou M. Li C. (2011). Cancer theranostics with near-infrared light-activatable multimodal nanoparticles. Acc. Chem. Res. 44 (10), 947–956. 10.1021/ar200022e 21848277 PMC3196765

[B30] Mohan R. (2010). Green bismuth. Nat. Chem. 2 (4), 336. 10.1038/nchem.609 21124518

[B31] Nikfarjam M. Muralidharan V. Malcontenti-Wilson C. Christophi C. (2005). The apoptotic response of liver and colorectal liver metastases to focal hyperthermic injury. Anticancer Res. 25 (2B), 1413–1419. 15865099

[B32] Ohtsuki C. Kokubo T. Yamamuro T. (1992). Mechanism of apatite formation on CaO SiO2P2O5 glasses in a simulated body fluid. J. Non-Crystalline Solids 143, 84–92. 10.1016/s0022-3093(05)80556-3

[B33] Pazarçeviren A. E. Tahmasebifar A. Tezcaner A. Keskin D. Evis Z. (2018). Investigation of bismuth doped bioglass/graphene oxide nanocomposites for bone tissue engineering. Ceram. Int. 44 (4), 3791–3799. 10.1016/j.ceramint.2017.11.164

[B34] Peng M. Dong G. Wondraczek L. Zhang L. Zhang N. Qiu J. (2011). Discussion on the origin of NIR emission from Bi-doped materials. J. Non-Crystalline Solids 357 (11-13), 2241–2245. 10.1016/j.jnoncrysol.2010.11.086

[B35] Peng M. Zollfrank C. Wondraczek L. (2009). Origin of broad NIR photoluminescence in bismuthate glass and Bi-doped glasses at room temperature. J. Phys. Condens. Matter 21 (28), 285106. 10.1088/0953-8984/21/28/285106 21828512

[B36] Prosecká E. Rampichová M. Litvinec A. Tonar Z. Kralickova M. Vojtova L. (2015). Collagen/hydroxyapatite scaffold enriched with polycaprolactone nanofibers, thrombocyte-rich solution and mesenchymal stem cells promotes regeneration in large bone defect *in vivo* . J. Biomed. Mater. Res. Part A 103 (2), 671–682. 10.1002/jbm.a.35216 24838634

[B37] Qi J. Shi D. Zhao J. Jiang X. (2008). Stable structures and electronic properties of the oriented Bi nanowires and nanotubes from first-principle calculations. J. Phys. Chem. C 112 (29), 10745–10753. 10.1021/jp801735g

[B38] Rustom L. E. Boudou T. Lou S. Pignot-Paintrand I. Nemke B. W. Lu Y. (2016). Micropore-induced capillarity enhances bone distribution *in vivo* in biphasic calcium phosphate scaffolds. Acta Biomater. 44, 144–154. 10.1016/j.actbio.2016.08.025 27544807 PMC5045872

[B39] Sun Y. Zhao Z. Dong F. Zhang W. (2015). Mechanism of visible light photocatalytic NO_x_ oxidation with plasmonic Bi cocatalyst-enhanced (BiO)_2_CO_3_ hierarchical microspheres. Phys. Chem. Chem. Phys. 17 (16), 10383–10390. 10.1039/c4cp06045h 25765222

[B40] Tian Q. Hu J. Zhu Y. Zou R. Chen Z. Yang S. (2013). Sub-10 nm Fe_3_O_4_@Cu_2–x_S core–shell nanoparticles for dual-modal imaging and photothermal therapy. J. Am. Chem. Soc. 135 (23), 8571–8577. 10.1021/ja4013497 23687972

[B41] Valerio P. Pereira M. M. Goes A. M. Leite M. (2004). The effect of ionic products from bioactive glass dissolution on osteoblast proliferation and collagen production. Biomaterials 25 (15), 2941–2948. 10.1016/j.biomaterials.2003.09.086 14967526

[B42] Velasco-Arias D. Zumeta-Dube I. Diaz D. Santiago-Jacinto P. Ruiz-Ruiz V. F. Castillo-Blum S. E. (2012). Stabilization of strong quantum confined colloidal bismuth nanoparticles, one-pot synthesized at room conditions. J. Phys. Chem. C 116 (27), 14717–14727. 10.1021/jp304170k

[B43] Von Maltzahn G. Park J-H. Agrawal A. Bandaru N. K. Das S. K. Sailor M. J. (2009). Computationally guided photothermal tumor therapy using long-circulating gold nanorod antennas. Cancer Res. 69 (9), 3892–3900. 10.1158/0008-5472.can-08-4242 19366797 PMC2712876

[B44] Wang L. Long N. J. Li L. Lu Y. Li M. Cao J. (2018). Multi-functional bismuth-doped bioglasses: Combining bioactivity and photothermal response for bone tumor treatment and tissue repair. Light Sci. Appl. 7 (1), 1–13. 10.1038/s41377-018-0007-z 30839587 PMC6106990

[B45] Wang Y. Wu Y. Liu Y. Shen J. Lv L. Li L. (2016). BSA-mediated Synthesis of bismuth sulfide nanotheranostic agents for tumor multimodal imaging and thermoradiotherapy. Adv. Funct. Mater. 26 (29), 5335–5344. 10.1002/adfm.201601341

[B46] Xiao Z. Xu C. Jiang X. Zhang W. Peng Y. Zou R. (2016). Hydrophilic bismuth sulfur nanoflower superstructures with an improved photothermal efficiency for ablation of cancer cells. Nano Res. 9 (7), 1934–1947. 10.1007/s12274-016-1085-y

[B47] Xie H. Li Z. Sun Z. Shao J. Yu X. F. Guo Z. (2016). Metabolizable ultrathin Bi_2_Se_3_Nanosheets in imaging-guided photothermal therapy. Small 12 (30), 4136–4145. 10.1002/smll.201601050 27329254

[B48] Yan X. Huang X. Yu C. Deng H. Wang Y. Zhang Z. (2006). The *in-vitro* bioactivity of mesoporous bioactive glasses. Biomaterials 27 (18), 3396–3403. 10.1016/j.biomaterials.2006.01.043 16504289

[B49] Yang Y. Wu H. Shi B. Guo L. Zhang Y. An X. (2015). Hydrophilic Cu_3_BiS_3_ nanoparticles for computed tomography imaging and photothermal therapy. Part. Part. Syst. Charact. 32 (6), 668–679. 10.1002/ppsc.201400238

[B50] Yu B. Pei P. Yu B. Li D. Zhang X. Huang J. (2017). Enhance the bioactivity and osseointegration of the polyethylene-terephthalate-based artificial ligament via poly(dopamine) coating with mesoporous bioactive glass. Adv. Eng. Mater. 19 (5), 1600708. 10.1002/adem.201600708

[B51] Yu X. Li A. Zhao C. Yang K. Chen X. Li W. (2017). Ultrasmall semimetal nanoparticles of bismuth for dual-modal computed tomography/photoacoustic imaging and synergistic thermoradiotherapy. ACS Nano 11 (4), 3990–4001. 10.1021/acsnano.7b00476 28395135

[B52] Zhao Z. Zhang W. Lv X. Sun Y. Dong F. Zhang Y. (2016). Noble metal-free Bi nanoparticles supported on TiO_2_ with plasmon-enhanced visible light photocatalytic air purification. Environ. Sci. Nano 3 (6), 1306–1317. 10.1039/c6en00341a

[B53] Zhi D. Yang T. O'hagan J. Zhang S. Donnelly R. F. (2020). Photothermal therapy. J. Control Release 325, 52–71. 10.1016/j.jconrel.2020.06.032 32619742

[B54] Zhu X. Feng W. Chang J. Tan Y. W. Li J. Chen M. (2016). Temperature-feedback upconversion nanocomposite for accurate photothermal therapy at facile temperature. Nat. Commun. 7 (1), 1–10. 10.1038/ncomms10437 PMC4742858 26842674

[B55] Zhu Y. Wu C. Ramaswamy Y. Kockrick E. Simon P. Kaskel S. (2008). Preparation, characterization and *in vitro* bioactivity of mesoporous bioactive glasses (MBGs) scaffolds for bone tissue engineering. Microporous Mesoporous Mater. 112 (1-3), 494–503. 10.1016/j.micromeso.2007.10.029

[B56] Zhu Y. Zhu R. Ma J. Weng Z. Wang Y. Shi X. (2015). *In vitro* cell proliferation evaluation of porous nano-zirconia scaffolds with different porosity for bone tissue engineering. Biomed. Mater. 10 (5), 055009. 10.1088/1748-6041/10/5/055009 26391576

